# Obesogenic Clusters Associated with Weight Status in Brazilian Adolescents of the *Movimente* School-Base Intervention

**DOI:** 10.3390/ijerph181910350

**Published:** 2021-09-30

**Authors:** Gabrielli Thais de Mello, Kelly Samara Silva, Thiago Sousa Matias, Maria Alice Altenburg de Assis, Adriano Ferreti Borgatto

**Affiliations:** 1 Research Center for Physical Activity and Health, Department of Physical Education, School of Sports, Federal University of Santa Catarina, Florianópolis 88040-900, Santa Catarina, Brazil; ksilvajp@gmail.com (K.S.S.); thiago.matias@ufsc.br (T.S.M.); 2 Post Graduate Program in Nutrition, Health Sciences Center, Federal University of Santa Catarina, Florianópolis 88040-900, Santa Catarina, Brazil; maria.assis@ufsc.br; 3 Department of Informatics and Statistics, School of Technology, Federal University of Santa Catarina, Florianópolis 88040-900, Santa Catarina, Brazil; afborgatto@hotmail.com

**Keywords:** adolescent, cluster analysis, diet, exercise, sedentary behavior

## Abstract

Background: the relationship between behavior clusters and weight status, mainly in low- and middle-income countries, remains unclear. This study aimed to examine the association between profiles of physical activity (PA), diet and sedentary behavior (SB) with weight status in adolescents from a southern Brazilian city, according to sex. Methods: data from the *Movimente* Intervention study were analyzed (*n* = 812 / mean age 13.0 years (sd 1.04). Data on SB hours per day, PA minutes per week and weekly consumption frequencies of fruits, vegetables, salty snacks, candies and soda were self-reported on the validated *Movimente* questionnaire. Classes of healthy and unhealthy behaviors were derived by latent class analysis. Logistic regression analysis was used to estimate the associations between adolescents’ weight status and classes. Results: two classes were identified for the whole sample and for boys and girls. All classes had high probabilities of engaging high time in SB. Male adolescents in the unhealthy class had low probabilities of being active and high probability of consuming a low-quality diet. In contrast, girls’ healthiest profile presented lower probabilities of being active compared to boys’ healthiest profiles. No association was found between weight status and classes. Conclusion: All classes had at least one unhealthy behavior, for both the whole sample, and for girls and boys. Girls’ profiles were unhealthier compared to boys’ profiles. Hence, it is recommended that intervention strategies to change behaviors need to be distinct according to sex, targeting more than one obesogenic behavior at the same time.

## 1. Introduction

The prevalence of obesity has increased 20% during the past three decades, based on data from 195 countries [1]. Three million schoolchildren in Brazil are obese or overweight [2]. Indeed, the etiology of obesity is multifactorial. Regarding behaviors factors there are evidences that physical inactivity, consumption of ultra-processed foods and sugar sweetened beverages, and spending high time on sedentary behaviors (SB) are established risk factors for obesity [3,4,5]. Thus, the number of studies assessing clusters of modifiable behaviors of lifestyle in early age are rising [4,6,7], since their coexistence is related to cumulative harmful effects to health [4,8,9], and the established behaviors in childhood track into adulthood [7].

Review studies on the clusters of physical activity (PA), diet and SB in youths reported that most children and adolescents presented mixed patterns, exhibiting one or two healthy behaviors combined with one or no unhealthy behaviors [4,7]. Patterns combine the intake of unhealthy foods items (e.g., soft drinks, junk foods) with SB, and clusters of high time practicing PA with high time in SB [4,7]. An European study conducted in 10 countries identified a large proportion of adolescents (*n* = 2084, 45% male) as being allocated in a cluster labelled inactive, high quality of diet [10]. Similar clusters were found in Brazilian adolescents characterized by practiced PA less than one day/week, high levels of SB (almost eight hours/day) and who eat unhealthy foods (more than three days/week) [11]. A Belgian study, conducted in children, identified the cluster inactive media-oriented unhealthy eaters, characterized by high Z-scores on screen-based media use and risk-related food consumption, but low Z-scores on leisure time PA and healthy food index [12]. In addition, there is evidence that these cluster behaviors differ according to sex, with boys being designated to clusters with high levels of PA [12,13,14,15]. In contrast, girls were more clustered in profiles characterized by better quality of diet and high screen time [4,15]. Such behavior clusters in adolescents are not well understood in low- and upper-middle-income countries; hence, there is need for investigation [4]. Understanding the correlation among different behaviors may help researchers to investigate the etiological factors of overweight or obesity in adolescents, contributing to the development of behavior change strategies by public health policies [16]. In addition, this can enforce the importance of promoting PA and healthy diet, and preventing SB in youths [17], and also help in the development of future interventions addressing a wide range of obesogenic behaviors rather than focusing on one behavior [7].

There are inconsistent findings regarding the association between behavior patterns and weight status. Some studies identified clusters characterized by either high SB and low levels of PA that are positively associated with being overweight [15,18,19], while others reported no association [10,13,20] or found an unexpected inverse association [15,19]. Considering cross-sectional and longitudinal analysis in American adolescents, girls presented higher odds of prevalence and incidences of obesity in the cluster labeled restrictive dieting & smoking, compared to the healthier cluster (school clubs & sports), and no association was found for boys [15]. In Brazilian schoolchildren aged 7–12 years (*n*= 5364), three dietary patterns were derived by latent profile analysis (traditional, monotonous and mixed), and none of these patterns were associated with overweightness or obesity for either boys or girls [21].

The majority of studies addressing cluster or latent class analysis on adolescent behaviors related to PA, diet and SB were conducted in high-income countries, and few studies evaluated these factors according to sex [4,15]. In order to identify clusters of PA, diet and SB according to sex and its association with weight status, a study was conducted among adolescents participating in the *Movimente* study in a Southern Brazilian city. The findings of this study will serve as a basis to assess the effectiveness of the intervention conducted by the *Movimente* study, and can contribute to the development of multicomponent interventions to increase PA levels, decrease SB and improve the quality of diet at the same time among adolescents. Furthermore, these data will aid to identify adolescent groups at higher risk of overweight and obesity, as well as to better understand how SB, PA and diet cluster together, as potential targets for interventions related to the school environment.

## 2. Material and Methods

### 2.1. Study Design and Population

This is a cross-sectional study using baseline data from the *Movimente* Intervention Program, carried out in 2017. This program aimed to increase PA and decrease SB among adolescents. The study was approved by the Federal University of Santa Catarina Ethics Committee (Protocol number 1.807.825 in 7 November 2016). The intervention was registered in the Clinical Trials Registry (NCT02944318), with the approval of the Brazilian National Research Ethics System (protocol number: 1.259.910).

The *Movimente* Program is a school-based cluster randomized controlled trial on adolescents (grades 7th to 9th). Information on intervention protocol, sample size and randomization can be found elsewhere [22]. All students from 7th to 9th grades from six schools were invited to participate in the study (*n* = 1427). In total, 999 adolescents provided written informed consent, and had free informed consent from parents (answer rate: 70%). Once students provided consent to participate in the study, they answered the *Movimente* questionnaire during regular school hours and anthropometric data were collected by trained professionals.

In this study, 921 students answered the questionnaire at baseline, and 859 and 861 had data on weight and height, respectively.

### 2.2. Cluster Variables

#### 2.2.1. Physical Activity

PA was assessed using the Self-Administered Physical Activity Checklist previously validated for Brazilian adolescents [23]. Adolescents reported PA practiced in a typical week in school and out of school, and also provided details on their weekly frequency and daily duration of 22 activities (with an option for record other activities). The total volume of PA in minutes for week was computed and split into three categories (<300, 300 to 419, ≥420 min/week) [24]].

#### 2.2.2. Sedentary Behavior

SB was assessed with four questions previously validated for Brazilian adolescents [25]. Adolescents reported the number of hours per day (week and weekend) spent watching television and playing videogames/computer games. Based on the time spent per activity obtained from the answers to the questions, the total time in SB was calculated for each screen device, using the following equation: [(TV week × 5) + (TV weekend × 2)/7] [26]. Thus, SB was obtained using the total screen time in hours per day, summing up the time in each screen, which was further categorized as follows: <2 h, 2 to 4 h and >4 h per day [24].

#### 2.2.3. Dietary Patterns

Using a standardized questionnaire [22,25], adolescents reported the weekly frequency of eating the following food items (one to seven days) in the last week: fruits, vegetables, salty snacks, candies and soda. Principal Component Analysis (PCA) was performed to identify the dietary patterns. To assess the suitability of the data for PCA, the Bartlett’s Test of Sphericity and the Kaiser-Meyer-Olkin test of sampling adequacy (KMO) were used, and values *p* < 0.05 and *p* > 0.60 were acceptable, respectively [27]. To obtain independence of the components, there is no need to perform orthogonal rotation. The number of components were decided with Kaiser criterion and the screen plot (eigenvalues >1) [27]. Factor loadings for each component represented the correlations of each predictor, with the dietary pattern score and higher absolute values indicating that the predictor contributes mostly to the construction of a particular component (see Supplementary Material Table S1). Two components were identified, and the food items in each component were scored and divided by the number of variables present in each component (2 and 3). The dietary components (patterns) were named according to the characteristics of the behaviors retained that resulted in two dietary patterns: fruit and vegetables (F&V); and sugar, salty snacks and sodas (SSS).

### 2.3. Weight Status

Height and weight were measured based on international standardization [28]. Anthropometric measurements were performed by trained researchers and taken from lightly dressed barefoot students. Height was measured with a portable stadiometer to the nearest 0.1 cm (Alturaexata^®^, Belo Horizonte, Brazil), with the subject standing up straight with feet together, head in Frankfort plane and arms hanging freely. Weight was measured using an electronic scale to the nearest 0.1 kg (Welmy^®^, Santa Bárbara d’Oeste, Brazil).

Body mass index (BMI) was computed as weight (kg)/height (m²). Age and sex-specific BMI z-scores were calculated according to the World Health Organization (WHO) [29]. Weight status was categorized as overweight including obesity (BMI z-score for age ≥ +1.0) and non-overweight (thinness and normal weight) (BMI z-score for age < +1). In the total sample, thinness prevalence was 2.6% (2.9% for boys and 2.3% for girls). Meanwhile, the prevalence of obesity was 12% in the total sample (14.8% for boys and 9.5% for girls).

### 2.4. Sociodemographic Variables

Sociodemographic variables included sex, age (years) and maternal level of education (non-educated, elementary school [low], high school [medium] and higher than high school education [high]). The students reported sociodemographic variables.

### 2.5. Statistical Analysis

Participants’ characteristics were described using means and standard deviations for quantitative variables and relative and absolute frequencies for qualitative variables. Comparisons of prevalence of overweight, including obesity vs. non-overweight between classes of behavior and between sexes, were performed using the chi-square test.

Latent Class Analysis (LCA) was performed in Software R, using the poLCA package. LCA is an optimal strategy for examining varying groupings based on an individual’s patterns of responses to multiple categories variables [30]. Latent class models were performed using four aforementioned health behaviors: (1) total PA (minutes per week); (2) weekly frequency of consuming healthy foods; (3) weekly frequency of consuming unhealthy foods; and (4) hours per day of SB. Models with two (model 1) to six classes (model 5) were performed, considering the whole sample and stratified by sex. Models were compared using Bayesian Information Criterion (BIC), Akaike Information Criterion (AIC), Consistent Akaike Information Criterion (CAIC), Adjusted Bayesian Information Criterion (ABIC) and the Log-Likelihood ratio [30]. Models with lower values of criteria were considered the best fit and parsimonious model. All participants were assigned to the class in which they had the highest posterior probability of membership. Class’s results were expressed as item-response probabilities for each behavior.

Statistical analyses for association were conducted using Stata Statistical software, version 15 (Stata Inc., College Station, TX, USA). Logistic regression adjusted by age and maternal education was used to verify the association of clusters with weight status (non-overweight/overweight, including obesity). Values were expressed as odds ratio (OR) and 95% confidence intervals (95% CI). The least healthy cluster was the reference category.

## 3. Results

From 921 students that answered the questionnaire, 109 students did not report some behavior variables (PA, diet or SB), 140 did not have weight and/or height measurements and 115 did not report maternal level education. Latent class analysis was performed for 88.7% of the sample (*n* = 812 [(boys = 386; girls *n* = 426)]), and association analysis was performed for 79.4% (*n* = 731). Table 1 shows sample characteristics for the total sample and by sex. The mean age of the participants was 13.0 years (sd 1.04), while 53.08% were girls, and 34.4% were overweight or obese (21.75% were overweight and 12.04% were obese). On the whole sample, about 51% of the students reported 420 min or more of weekly PA, although there was difference between sexes, judging by the CI: a higher proportion of boys reported 420 min or more of weekly PA, compared to girls (62.4% vs. 42.3%). SB (>4 h/day) was also reported by a higher proportion of boys compared to girls (65% vs. 47.7%). Alternatively, the consumption frequency of F&V (4 or more times per week) was reported by a higher proportion of girls compared to boys (40.6% vs. 30.6%). The consumption frequencies (two or more times/week) of foods sources containing sugar and salt and sodas (SSS) were reported by about 50% of the sample, and virtually no difference was observed between sexes.

### Latent Class Profiles

**Figure 1:**
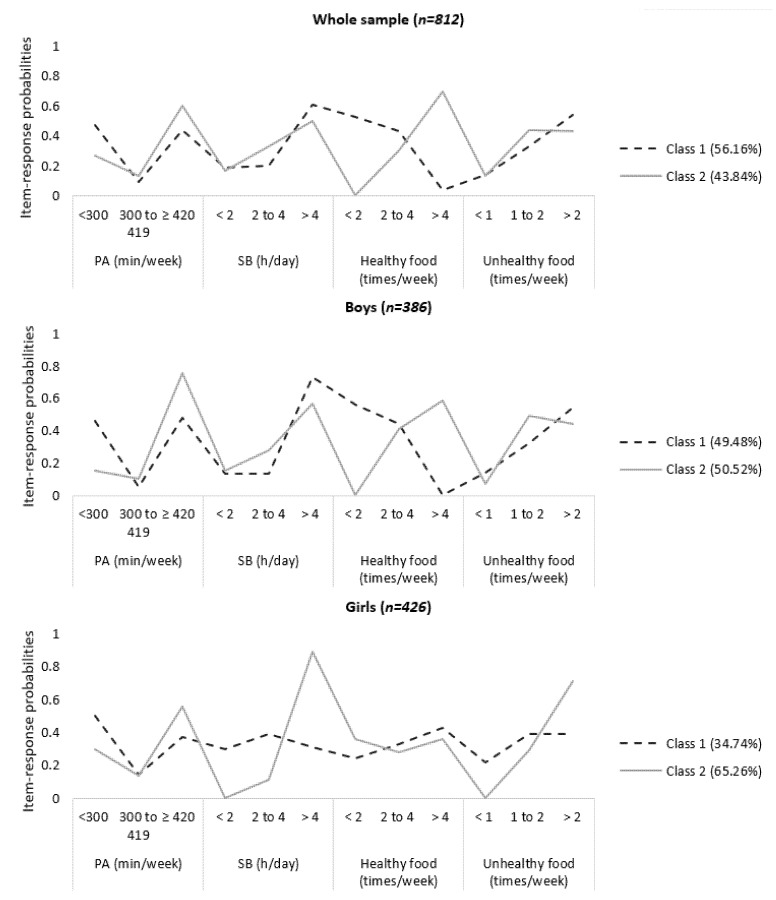
Prevalence and item-response probabilities of each risk behavior for each class in Brazilian adolescents. Note: PA: physical activity; SB: sedentary behavior. Healthy food: fruit and vegetables. Unhealthy food: sugar, salt and soda.

The model fit for the models examining 2–6 latent classes is presented in Supplementary Material Table S2. The 2-class model was selected as the best-fitting model for the whole sample, for both boys and girls, since it is conceptually more meaningful than the others and had the lowest values for BIC, ABIC and CAIC. Clusters characteristics and item-response probabilities are shown in Supplementary Material Table S3 and Figure 1.

**Figure 2:**
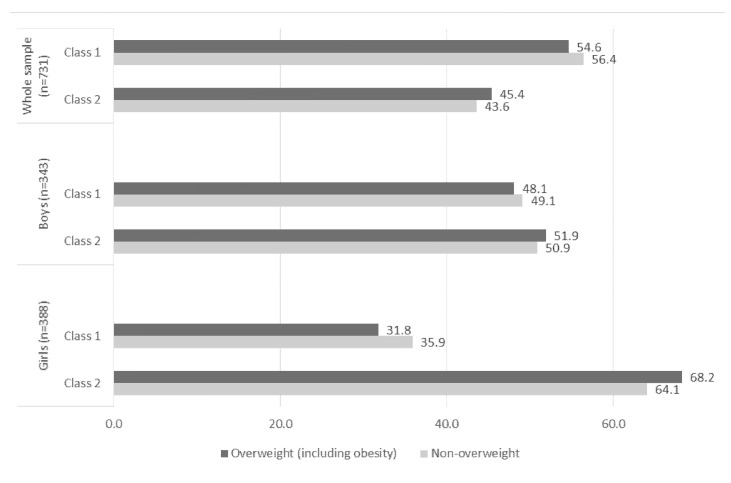
Weight status according to classes of behaviors in Brazilian adolescents. Florianópolis, Santa Catarina, Brazil, 2017. Note: chi-square test indicated no statistical significant differences.

Figure 2 shows the weight status of the students according to classes of behaviors. There were no significant differences between weight status and classes in all stratums (whole sample, boys and girls).

Total sample. Class 1 (56.16% of the students) was the unhealthiest, and was mainly characterized by the high probability of practicing PA <300 min per week, spending >4 h per day engaged in SB, consuming F&V <2 times per week and SSS >2 times per week. On the other hand, class 2 (43.84% of the students) were characterized by the high probability of practicing PA ≥420 min per week, spending >4 h per day engaged in SB and consuming >4 and 1–2 times for week of F&V and SSS, respectively.

Boys. Class 2 was composed by 50.52% of boys, who had high probabilities of practicing PA ≥420 min weekly, consuming F&V >4 times per week, spending >4 h per day engaged in SB and consuming 1 to 2 times per week of SSS. Class 1 (unhealthiest) comprised 49.48% of boys whose behaviors were characterized by low probabilities of practicing PA ≥420 min weekly and consuming F&V >4 times per week. In addition, they had high probabilities of spending >4 h per day engaged in SB, and consuming SSS >2 times per week.

Girls. Class 2 included a higher proportion of girls (65.26%), compared to Class 1. Girls in this class had high probabilities of practicing ≥420 min per week of PA, spending >4 h per day of engaged in SB and consuming SSS >2 times per week. Members of this class also presented low probabilities of consuming F&V >4 times per week. Class 1 comprised 34.74% of girls, which was characterized by higher probabilities of practicing <300 min per week of PA, spending 2 to 4 h per day engaged in SB, consuming >4 per week of F&V and >2 and 1 to 2 times per week of SSS.

Table 2 shows the relationship between weight status (non-overweight vs. overweight, including obesity) and classes of behaviors patterns for the whole sample. In the adjusted model, no significant associations were found.

## 4. Discussion

This study examined the patterns of PA, diet and SB and assessed its associations with weight status among Brazilian adolescents. Due to country-specific differences in food intake, PA and SB, the present study provides valuable information on clusters of these behaviors among adolescents from an upper-middle-income country. Furthermore, data from high-income countries showed that the prevalence of overweight and obesity among young people have plateaued. In contrast, data from medium- to low-income countries indicates that the prevalence is still rising [31].

Two classes were identified for all stratum (whole sample and for boys and girls). All classes had high probabilities of engaging high time in SB. No association was found between weight status and clusters. The unhealthiest profile (Class 1) was more prevalent among the participants, which is in line with previous studies [4,11]. Class 1 was predominantly characterized by low probability to practice PA for ≥420 min per week, low-quality diet and high time spent in SB. Nevertheless, classes characterized with high or low probabilities to practice PA still presented high probabilities of SB, corroborating with data from the literature where high time practicing PA coexists with high time spent in SB in the same cluster [4,11]. A Brazilian study reported that adolescents had frequent consumption of unhealthy foods, spent high time in SB and had low levels of PA [32]. Using data from 102,072 Brazilian students attending the ninth-grade of public and private schools and who completed the 2015 Brazilian National School-Based Adolescent Health Survey (PeNSE), Matias et al. identified three clusters, using two-steps cluster analysis: (1) health-promoting SB and diet (32.6% of the students); (2) health-promoting PA and diet (44.9%); and (3) health-risk (22.5%) [11]. The latter cluster presented more unhealthy behaviors than our unhealthiest pattern (class 1). Similarities were found by several other studies [33,34,35], while PA practice was positively associated with healthy diet habits of high quality in some studies [33,36,37]. High probabilities of SB can be explained by compensatory health beliefs, wherein adolescents believe that the negative effects of indulgence in SB can be compensated by subsequent exercise or healthy diet [38].

More boys were allocated in the healthiest profile (Class 2) characterized by high time practicing PA and weekly frequency of consuming fruits and vegetables. The boys in the unhealthiest class had low probabilities of being active and consuming a low-quality diet. In contrast, the girls’ healthiest profile presented low probabilities of being active compared to boys’ healthiest class, corroborating with data from the literature [4,7,12,13]. In addition, both classes in boys and girls had higher probabilities of spending more than 2 h engaged in SB, a result similar to other studies where adolescents with healthier profile still engaged in at least one unhealthy behavior [4,11]. Boys and girls in both classes presented mixed profiles, characterized by at least two unhealthy behaviors combined with healthy behaviors.

Studies have identified that girls had the unhealthiest profiles and a higher presence in clusters with low levels of PA compared to boys [12,13]. These differences can be explained in accordance to how boys and girls spend their time; girls are more likely to socialize and undertake domestic tasks, while boys practice PA [39]. Furthermore, girls are less motivated and had low willingness to practice PA as it results in being dirty and sweaty [40].

These findings enforce the importance of interventions to promote PA and the consumption of healthy foods, as well as reduce SB in youths [17], once they are established risk factors with a cumulative harmful effect to health. In this sense, it seems important to develop behavior change programs targeting multiple obesogenic behaviors at the same time, as once a behavior is changed, other healthy synergistic changes can also occur.

It is difficult to make direct comparisons between the present results and those of other studies, due to the different analytical methods used to derive clusters behaviors among adolescents, such as cluster k-means [10,14,19] and two-step cluster analyses [11,18,20], while LCA was used in the present study. Furthermore, distinct variables of PA, diet and SB were used to define profiles and others behaviors such us sleep duration, alcohol consumption and tobacco use were included in other surveys [4,7]. In some studies, weight and height were self-reported by children/adolescents or their parents, and different BMI references (e.g., WHO, International Obesity Task Force—IOTF) to define overweight and obesity were used [13,14,19,20].

Our results unexpectedly found no association between clusters and weight status, a finding that is not unique [4,13,14,15]. A possible explanation is that overweight adolescents tend to underreport their unhealthy behaviors [41]. Diet behaviors were assessed by questions regarding weekly frequencies of consumption of five food groups, and did not ask about portion sizes or quantities, and thus it was not possible to obtain the energy value of foods. We hypothesize that latent classes derived from short food questionnaires, based on weekly frequency of consumption rather than quantity, do not substantially drive body adiposity. In addition, there was coexistence of healthy and unhealthy behaviors at the same clusters. In contrast, a recent systematic review has shown that across clusters of obesogenic behaviors, derived by latent class analysis, those characterized by low consumption of fruit and vegetables and the high consumption of fatty foods, sugar snack foods, sweets, chips and fries, low PA (<1 h each day) and high SB (screen time and TV >2 h/day) and sleep time (<10 h/day) were positively associated with overweight and/or obesity in children and adolescents [42].

There are limitations that must be considered when interpreting the study results. Data was self-reported, which has well-known disadvantages (e.g., under- or over-reporting), and make it impossible to explore the domain of variables, intensities of PA and energy intake, although all questionnaires used were validated. Objective measurements, such as an accelerometer, are more accurate to determine the amount of PA and SB compared to questionnaires [43]. However, the use of accelerometers in population surveys requires a lot of financial resources and adequate technical support, making it difficult in studies conducted in low- and middle-income countries. On the other hand, our study has some strength. The study aids to fill research gaps, since the sample was composed of Brazilian adolescents and investigated cluster behaviors in an upper-middle income country [4]. In addition, the use of latent class analysis allowed the identification of exclusive subgroups of individuals who share similar behaviors. In order to provide meaningful insights in future intervention programs for PA improvement and SB reduction, it is also relevant to investigate specific domains, like school, sport and leisure time, in which PA takes place [44]. Relative to diet behavior, the investigation of the school (e.g., presence of canteens, school meals’ menus and the availability of fresh water) and home environment (i.e., availability of healthy and unhealthy foods) are valuable topics to better understand the drivers of food intake behavior. Physical activity, sedentary time and food intake differ on school days compared to the weekend, and thus it is important to distinguish these behaviors according to the day of the week in future research [45].

## 5. Conclusions

The classes of PA, diet and SB in Brazilian adolescents can be described in two profiles characterized by at least one unhealthy behavior, with all classes presenting high probabilities to spend more than 2 h engaged in SB. No association between classes and weight status was observed. Once the classes were predominantly characterized as unhealthy behaviors and the obesity etiology is multifactorial, results suggest the development of multicomponent strategies, which focus on a wide range of obesogenic behaviors, enable the shift to a healthier lifestyle. Thus, programs should consider different actions according to sex, promoting PA for girls and a balanced diet and SB for both sexes.

## Figures and Tables

**Table 1:** Sample characteristics. Florianópolis, Santa Catarina, Brazil, 2017.

Variables	Total Sample (*n* = 731)		Boys (*n* = 343)		Girls (*n* = 388)	
Age (Mean ± sd)	13.0 ± 1.04		13.1 ± 1.06		13.1 ± 1.02	
	% (95% CI)					
**Maternal level education**						
Low	24.2	(21.2, 27.4)	21.8	(17.7, 26.5)	26.2	(22.1, 30.9)
Medium	27.6	(24.5, 30.9)	27.9	(23.4, 33.0)	27.3	(23.0, 31.9)
High	18.3	(15.6, 21.3)	19.2	(15.3, 23.7)	17.5	(14.0, 21.6)
Unknown	29.8	(26.6, 32.2)	30	(26.2, 36.0)	28.8	(24.5, 33.5)
**PA (min/week)**						
<300	38.6	(35.4, 41.8)	30.1	(25.6, 34.8)	43.9	(39.2, 48.6)
300 to 419	10.6	(8.7, 12.7)	7.5	(5.2, 10.6)	13.9	(10.8, 17.4)
≥420	50.8	(47.5, 54.1)	62.4	(57.4, 67.1)	42.3	(37.6, 47.0)
**SB (h/day)**						
<2	18.3	(15.8, 20.9)	14.3	(11.0, 18.1)	21.4	(17.7, 25.5)
2 to 4	25.8	(23.0, 28.8)	20.7	(16.9, 25.0)	31.0	(26.7, 35.5)
>4	55.9	(52.6, 59.1)	65.0	(60.1, 69.6)	47.7	(42.9, 52.4)
**F&V (times/week)**						
<2	27.5	(24.6, 30.4)	26.9	(22.7, 31.6)	27.5	(23.4, 31.9)
2 to 4	36.3	(33.1, 39.5)	42.5	(37.6, 47.4)	31.9	(27.6, 36.5)
>4	36.3	(33.1, 39.5)	30.6	(26.1, 35.3)	40.6	(36.0, 45.3)
**SSS (times/ week)**						
<1	12.9	(10.8, 15.2)	10.4	(7.68, 13.8)	15.5	(12.3, 19.2)
1 to 2	38.1	(34.9, 41.3)	40.9	(36.1, 45.9)	35.9	(31.4, 40.6)
>2	49.0	(45.7, 52.3)	48.7	(43.7, 53.7)	48.6	(43.8, 53.3)
**Weight status**						
Non-overweight	65.7	(62.4, 68.7)	63.1	(58.3, 67.6)	68.0	(63.5, 72.2)
Overweight (including obesity)	34.4	(31.2, 37.5)	36.9	(32.3, 41.6)	32.0	(27.7, 36.4)

CI: confidence interval. sd: standard deviation. Maternal education: Low (non-educated, elementary school); Medium (high school); High (higher than high school education); and unknown (adolescent did not know maternal education). PA: physical activity. SB: sedentary behavior. F&V: fruit and vegetables. SSB: sugar, salt and soda.

**Table 2:** Latent classes associated with weight status (*n* = 731). Florianópolis, Santa Catarina, Brazil, 2017.

Classes	Overweight (Including Obesity)			
	Crude		Adjust	
	OR	95% CI	OR	95% CI
Whole sample				
Class 1	1		1	
Class 2	1.11	(0.82, 1.51)	1.15	(0.84, 1.57)
Boys				
Class 1	1		1	
Class 2	1.19	(0.76, 1.86)	1.24	(0.78, 1.97)
Girls				
Class 1	1		1	
Class 2	1.27	(0.80, 2.02)	1.24	(0.77, 1.99)

Category non-overweight was reference. Class 1, considered unhealthiest, was reference. OR = odds ratio; CI 95% = 95% confidence interval. Models adjusted by age and maternal education.

## Data Availability

Data sharing is not applicable to this article.

## Supplementary Materials

The following are available online at https://www.mdpi.com/article/10.3390/ijerph181910350/s1, Table S1: Factor loading to dietary patterns resulting from Principal Component Analysis in Brazilian adolescents (*n* = 812), Table S2: Latent class models parameters, Table S3: Prevalence and item-response probabilities for the 2 latent class model of physical activity, diet and sedentary behavior of Brazilian adolescents.
