# Supplementary material for: Obesogenic Clusters Associated with Weight Status in Brazilian Adolescents of the Movimente School-Base Intervention
Source: Int J Environ Res Public Health. 2021 Sep 30;18(19):10350. doi: 10.3390/ijerph181910350 (PMC8507682; doi:10.3390/ijerph181910350)
Supplement: Supplementary file 1 [file ijerph-18-10350-s001.zip › ijerph-1346380-supplementary.pdf]

Supplementary Materials

Table S1. Factor loading to dietary patterns resulting from Principal Component Analysis in Brazilian adolescents (n = 812).

Variables	Components	
	SSB	F&V
Fruit		0.68
Vegetable		0.64
Crisps	0.58	
Candies	0.53	
Soda	0.54	

Kaiser Meyer Olkin= 0.61. Bartlett test of sphericity $p < 0.001$). F&V = fruit and vegetables. SSB = sugar, salt and beverages (soda).

Table S2. Latent class models parameters

Model	Log Likelihood	Degrees of Freedom	BIC	ABIC	CAIC	Likelihood Ratio
**Total sample**						
Model 1	−3236.52	63	6586.94	6532.96	6603.94	99.9405
Model 2	−3220.93	54	6616.04	6533.47	6642.04	68.7430
Model 3	−3212.59	45	6659.67	6548.52	6694.67	52.0739
Model 4	−3204.84	36	6704.45	6564.72	6748.45	36.5628
Model 5	−3200.62	27	6756.32	6588.01	6809.32	28.1386
**Boys**						
Model 1	−1431.18	63	2963.61	2909.67	2980.61	103.42
Model 2	−1422.52	54	2999.90	2917.40	3025.90	86.11
Model 3	−1415.49	45	3039.42	2928.37	3074.42	72.03
Model 4	−1409.41	36	3080.88	2941.27	3124.88	59.88
Model 5	−1401.88	27	3119.41	2951.25	3172.41	44.81
**Girls**						
Model 1	−1751.08	63	3605.09	3551.14	3622.09	83.57
Model 2	−1742.81	54	3643.04	3560.53	3669.04	67.03
Model 3	−1737.05	45	3686.01	3574.95	3721.01	55.51
Model 4	−1731.54	36	3729.47	3589.84	3773.47	44.48
Model 5	−1727.00	27	3774.89	3606.70	3827.89	35.41

BIC: Bayesian information criterion; ABIC: Adjusted Bayesian information criterion, CAIC: Consistent Akaike information criterion.

Table S3. Prevalence and item-response probabilities for the 2 latent class model of physical activity, diet and sedentary behavior of Brazilian adolescents.

	Total Sample (*n* = 812)		Boys (*n* = 386)		Girls (*n* = 426)	
	**Class 1**	**Class 2**	**Class 1**	**Class 2**	**Class 1**	**Class 2**
Latent Class n (%)	456 (56.16)	356 (43.84)	191 (49.48)	195 (50.52)	148 (34.74)	278 (65.26)
Item-response probabilities						
PA (minutes/week)						
<300	0.47	0.27	0.46	0.15	0.50	0.30
300 to 419	0.09	0.13	0.05	0.10	0.14	0.14
≥420	0.44	0.60	0.48	0.76	0.37	0.56
SB (hours/day)						
<2	0.19	0.17	0.13	0.15	0.30	0.00
2 to 4	0.20	0.33	0.13	0.28	0.39	0.11
>4	0.61	0.50	0.73	0.57	0.31	0.89
F&V (times/week)						
<2	0.53	0.00	0.56	0.00	0.24	0.36
2 to 4	0.43	0.30	0.44	0.41	0.33	0.28
>4	0.04	0.70	0.00	0.59	0.43	0.36
SSB (times/ week)						
< 1	0.14	0.13	0.14	0.07	0.22	0.00
1 to 2	0.33	0.44	0.32	0.49	0.39	0.29
>2	0.54	0.43	0.54	0.44	0.39	0.71

PA = physical activity. SB = sedentary behaviors. F&V = fruit and vegetables. SSB = sugar, salt and beverages.
